# Irrigation fluid temperature modulates coagulation and endothelial biomarkers in patients undergoing TURP

**DOI:** 10.5937/jomb0-61030

**Published:** 2026-01-28

**Authors:** Min Gu, Yanqiu Xu

**Affiliations:** 1 Suzhou Hospital of Integrated Traditional Chinese and Western Medicine, China; 2 Suzhou Hospital of Integrated Traditional Chinese and Western Medicine, Department of Urothoracic Surgery, China

**Keywords:** activated partial thromboplastin time (APTT), platelet function, endothelin-1, coagulation biomarkers, hypothermia, aktivirano parcijalno tromboplastinsko vreme (APTT), funkcija trombocita, endotelin-1, biomarkeri koagulacije, hipotermija

## Abstract

**Background:**

The temperature of irrigation fluid is a crucial yet frequently neglected factor affecting perioperative hemostasis during transurethral resection of the prostate (TURP). Although the clinical implications of hypothermia are well-documented, its biochemical effects on coagulation processes and endothelial markers are not fully elucidated.

**Methods:**

Ninety patients with benign prostatic hyperplasia undergoing TURP were randomized into three groups based on irrigation fluid temperature: hypothermia (24-26 °C), mild hypothermia (28-35 °C), and preheating (36-37 °C). Peripheral blood was collected preoperatively and 6 h postoperatively to evaluate coagulation and endothelial indices, including prothrombin time (PT), activated partial thromboplastin time (APTT), platelet count (PLT), platelet aggregation rate (Pagt), and endothelin-1 (ET-1).

**Results:**

Hypothermia significantly prolonged APTT and reduced PLT and Pagt compared with preheating (P &lt; 0.05), indicating impaired intrinsic pathway activity and platelet dysfunction. PT remained unchanged across groups. ET-1 levels decreased in both hypothermia and preheating groups, with the greatest reduction in preheating, suggesting differential endothelial responses. Patients in the preheating group exhibited preserved coagulation stability and fewer adverse events (6.7%) compared with hypothermia (33.3%) and mild hypothermia (23.3%) groups (P = 0.038).

**Conclusions:**

Hypothermic irrigation significantly prolonged APTT, reduced platelet count, and impaired aggregation capacity, while preheating maintained hemostatic homeostasis.

## Abstract (sr)

**Uvod:**

Temperatura irigacione tečnosti je ključni, ali često zanemareni faktor koji utiče na perioperativnu hemostazu tokom transuretralne resekcije prostate (TURP). Iako su kliničke implikacije hipotermije dobro dokumentovane, njeni biohemijski efekti na procese koagulacije i endotelne markere nisu u potpunosti razjašnjeni.

**Metode:**

Devedeset pacijenata sa benignom hiperplazijom prostate koji su podvrgnuti TURP-u su randomizovani u tri grupe na osnovu temperature irigacione tečnosti: hipoter-mija (24-26 °C), blaga hipotermija (28-35 °C) i prethodno zagrevanje (36-37 °C). Periferna krv je prikupljena preop-erativno i 6 sati posle operacije radi procene koagulacionih i endotelnih indeksa, uključujući protrombinsko vreme (PT), aktivirano parcijalno tromboplastinsko vreme (APTT), broj trombocita (PLT), brzinu agregacije trombocita (BAgt) i endotelin-1 (ET-1).

**Rezultati:**

Hipotermija je značajno produžila APTT i smanjila PLT i Pagt u poređenju sa prethodnim zagrevanjem (P &lt; 0,05), što ukazuje na oštećenu aktivnost unutrašnjeg puta i disfunkciju trombocita. PT je ostao nepromenjen u svim grupama. Nivoi ET-1 su se smanjili i u grupi sa hipotermijom i u grupi sa prethodnim zagrevanjem, sa najvećim smanjenjem kod prethodnog zagrevanja, što ukazuje na diferencijalne endotelne odgovore. Pacijenti u grupi sa prethodnim zagrevanjem pokazali su očuvanu stabilnost koagulacije i manje neželjenih događaja (6,7%) u poređenju sa grupama sa hipotermijom (33,3%) i blagom hipotermijom (23,3%) (P = 0,038).

**Zaključak:**

Hipotermna irigacija je značajno produžila APTT, smanjila broj trombocita i oštetila kapacitet agregacije, dok je prethodno zagrevanje održavalo hemostatsku homeostazu.

## Introduction

Benign prostatic hyperplasia (BPH) is a prevalent condition in older men, especially after age 50, causing urinary issues like frequency, urgency, nocturia, and incomplete emptying, which affect quality of life. Transurethral resection of the prostate (TURP) is the preferred surgical treatment due to its minimally invasive nature and effectiveness. Post-surgery, continuous bladder irrigation with 0.9% sodium chloride is used to prevent clots, keep the catheter clear, and reduce irritation. Recent studies suggest that the irrigation fluid's temperature significantly impacts perioperative safety and recovery [1].

Hypothermia irrigation fluid has been reported to exacerbate bladder spasm by stimulating the detrusor muscle, and more importantly, to impair hemostasis by inducing coagulation dysfunction [2]. The mechanisms involve decreased thrombin activity, changes in platelet aggregation, and fibrinolytic system activation. Studies indicate that hypothermic irrigation leads to nearly three times more postoperative blood loss and higher transfusion needs compared to normothermic solutions. On the other hand, overly warm fluids can cause local hyperemia, delay wound healing, and increase bleeding risk. Therefore, finding the ideal irrigation fluid temperature is crucial for balancing mucosal protection and hemostatic stability.

Although clinical observations exist, the biochemical and hematological changes causing bleeding due to hypothermia during TURP are not well understood. Coagulation indices like PT, APTT, PLT, and Pagt, along with vascular markers like ET-1, offer important insights into hemostatic and vascular functions. Systematically assessing these parameters at various irrigation temperatures could clarify the mechanisms of hypothermia-related coagulation issues and highlight their diagnostic and predictive potential [3].

This study examined how the temperature of irrigation fluid affects coagulation and endothelial function in TURP patients with BPH. It aims to offer biochemical evidence for thermal management strategies to reduce postoperative bleeding and enhance patient outcomes.

## Materials and methods

### General information

This study was approved by the institutional Ethics Committee (Ethics Number: (20223131KY)). Written informed consent was obtained from all patients or their legal guardians. Ninety patients diagnosed with benign prostatic hyperplasia (BPH) and classified as ASA grade II-III who underwent elective TURP were enrolled. Patients were allocated into three groups (n = 30 each) based on the intraoperative irrigation fluid temperature: the hypothermia group (24-26°C), the mild hypothermia group (28-35°C), and the preheating group (36-37°C). Patient age ranged from 52 to 79 years (mean ± SD, 68.2 ± 10.0 years). The disease duration was 2-4 years (3.2 ± 0.4 years), prostate weight was 22-146 g (63.1 ± 22.1 g), and prostate volume ranged from 35 to 94 cm^2^ (61.7 ± 9.2 cm^2^). The average operative time was 39.6 ± 12.8 min. The indwelling urinary catheter balloon volume ranged from 12.5 to 20 mL (mean, 16.2 ± 3.7 mL), and the catheter remained in situ for 2-5 days (3.3 ± 1.2 days). Digital rectal examination using Rous criteria classified 45 patients with grade I hyperplasia, 35 with grade II, 5 with grade III, and 5 with grade IV.

### Inclusion and exclusion criteria

Inclusion criteria for the study comprised patients exhibiting pronounced symptoms of bladder irritation and bladder outlet obstruction attributable to benign prostatic hyperplasia (BPH), characterized by an International Prostate Symptom Score (IPSS) of 12 or higher, or confirmed obstruction via urodynamic assessment [4]. Eligible patients demonstrated a maximum urinary flow rate (Qmax) of less than 10 mL/s, accompanied by a post-void residual urine volume exceeding 150 mL, following the exclusion of non-obstructive etiologies such as neurogenic bladder. Furthermore, additional criteria encompassed imaging evidence indicative of obstruction-related complications or renal impairment.

Exclusion criteria for the study encompassed the following conditions: uncontrolled hypertension, defined as a systolic blood pressure exceeding 180 mmHg or a diastolic pressure exceeding 110 mmHg; unstable angina; myocardial infarction or stroke within the preceding six months; diabetes mellitus complicated by ketoacidosis, hyperosmolar hyperglycemic state, or glycated hemoglobin (HbA1c) levels greater than 9.0% accompanied by significant glucose fluctuations. Additional exclusion criteria included hepatic dysfunction classified as Child-Pugh grade B or higher, severe coagulation abnormalities (international normalized ratio [INR] greater than 1.5 or platelet count below 50 X 10^9^/L), chronic renal failure with an estimated glomerular filtration rate (eGFR) below 30 mL/min/1.73 m^2^, or acute kidney injury. Participants with systemic bleeding disorders, such as hemophilia or thrombocytopenic purpura, those undergoing anticoagulation therapy with subtherapeutic INR, individuals with allergies to anesthetics, those classified as ASA grade IV or higher, and those with active urinary tract infections or concurrent urinary tract pathologies necessitating priority treatment were also excluded.

### Surgical and anesthetic methods

Operating room temperature was maintained at 24-26°C and humidity at 40-50%. All patients received intramuscular atropine (0.5 mg) 30 min before anesthesia. Baseline blood pressure, heart rate, and oxygen saturation were recorded using a Mindray monitor after 10 min of rest. Peripheral venous access was established, and 2 mL of venous blood was drawn for baseline testing. Continuous epidural anesthesia was administered at the L_2_-L interspace using 0.89% ropivacaine mesylate (Anhui Weierman Pharmaceutical Co., LTD; batch number 0809017) to achieve a sensory block from T -S . Sedatives were administered sparingly for anxious patients.

Intravenous infusion of compound sodium chloride and succinyl gelatin at 4-6 mL/(kg-h) was used, preheated to 35-37°C. Irrigation was performed using 5% glucose solution, with a mean total volume of approximately 20,000 mL. Core body temperature was continuously monitored using an anal temperature probe inserted 15 cm into the sigmoid colon. The irrigation bag was maintained 60-70 cm above the surgical field.

### Blood sampling and laboratory measurements

Peripheral venous blood (5 mL) was collected from each patient at two time points: preoperatively (within 30 min before anesthesia) and postoperatively (6 h after surgery). Samples were drawn into vacuum tubes containing 3.2% sodium citrate at a ratio of 9:1 (blood:anticoagulant), immediately mixed, and processed within 1 h of collection. Plasma was obtained by centrifugation at 2,500 X g for 15 min at room temperature.

The following laboratory parameters were determined:

### Prothrombin time (PT) and activated partial thromboplastin time (APTT):

Measured using a fully automated coagulation analyzer (Sysmex CA-7000, Sysmex Corporation, Kobe, Japan) with commercial reagents (Siemens Healthcare Diagnostics, Germany). PT was determined by the clotting method using thromboplastin reagent, and APTT was assessed using an activated cephalin reagent. Quality control was performed daily with normal and abnormal control plasmas provided by the manufacturer.

### Platelet count (PLT):

Determined using an automated hematology analyzer (Beckman Coulter LH 780, Beckman Coulter, USA). The analyzer employed impedance and optical detection methods for platelet quantification. Internal quality control was conducted according to Clinical and Laboratory Standards Institute (CLSI) guidelines.

### Platelet aggregation rate (Pagt):

Measured by light transmission aggregometry using an AggRAM Platelet Aggregometer (Helena Laboratories, USA). Platelet-rich plasma (PRP) was prepared by centrifuging whole blood at 150 X g for 10 min. Platelet-poor plasma (PPP) was obtained by centrifugation at 2,500 X g for 15 min. Aggregation was induced with 10 pmol/L adenosine diphosphate (ADP), and the maximal aggregation rate (%) was recorded.

### Endothelin-1 (ET-1):

Plasma ET-1 concentration was measured by enzyme-linked immunosorbent assay (ELISA) using a commercially available kit (R&D Systems, Minneapolis, MN, USA) according to the manufacturer's instructions. Absorbance was read at 450 nm with a reference wavelength of 570 nm using a microplate reader (BioTek Instruments, USA). The assay sensitivity was 0.5 pg/mL, and intra- and inter-assay coefficients of variation were <10%.

### Observation indicators

Primary laboratory indicators included PT, APTT, PLT, Pagt, and ET-1 levels. Secondary perioperative variables (e.g., irrigation time, bladder spasm duration) were recorded but were not the focus of the analysis.

### Statistical analysis

Statistical analysis was performed using SPSS26.0 (SPSSInc.,Chicago,USA). The included data all conformed to the normal distribution. Measurement data were expressed as mean ± standard deviation (x̄±s), and the variance f-test was used for comparisons between and within multiple groups. Counting data were expressed as rates (%), and the comparison between groups was performed using the χ^2^ test or Fisher's exact probability method. All hypothesis tests were conducted using two-sided tests, and the test level was set at α = 0.05. A P value <0.05 was considered statistically significant.

## Results

### Comparison of baseline and intraoperative data

Significant intergroup differences were observed in operative time (F = 19.251, P < 0.001) and anal temperature (F = 3.145, P = 0.047). The preheating group had the longest mean operative time (85.40 ± 10.41 min), which was significantly longer than that of the hypothermia group (76.16 ± 10.29 min) and the mild hypothermia group (78.17 ± 10.23 min). Anal temperature was also significantly higher in the preheating group (37.62 ± 1.42°C) compared with the hypothermia (36.28 ± 4.14°C) and mild hypothermia groups (36.12 ± 3.39°C). Other perioperative parameters, including total local anesthetic volume (7.46-8.39 mL), intraoperative infusion volume (816.42-838.22 mL), irrigation fluid temperature (27.49-36.42°C), room temperature (24.93-25.53°C), mean arterial pressure (119.69-123.62 mmHg), heart rate (78.2-82.2 beats/min), and SpO_2_ (96.45-97.53%), did not differ significantly among groups (P > 0.05) (Table 1).

**Table 1:** Comparison of test data of the three groups of patients.

Factors	hypothermia group<br>(30)	Mild hypothermia <br>group (30)	Preheating group<br>(30)	*F*	*P*
Operation time (min)	76 .16 ± 10 .29	78 .17 ± 10 .23	85 .40 ± 10 .41	19.251	< 0.001
Intraoperative Infusion volume (mL)	816 .65 ± 118 .20	816 .42 ± 106 .97	838 .22 ± 110 .13	0 .188	0 .828
Rinse fluid temperature (°C)	27 .49 ± 6 .28	29 .37 ± 4 .54	36 .42 ± 8 .22	0.177	0 .429
Room temperature (°C)	24 .98 ± 7 .51	24 .93 ± 6 .36	25 .53 ± 6 .56	0.246	0 .403
MAP (mmHg)	119 .69 ± 13 .36	120 .73 ± 10 .49	123 .62 ± 10 .41	0.876	0 .415
Heart Rate (times/min) SPO_2_ (%)	78 .2 ± 7 .6	79 .2 ± 8 .7	82 .2 ± 6 .3	1.321	0 .270
Anal temperature (°F)	96 .45 ± 10 .27	97 .39 ± 10 .21	97 .53 ± 10 .27	0.123	0 .885
Total local anesthetic (mL)	36 .28 ± 4 .14	36 .12 ± 3 .39	37 .62 ± 1 .42	3.145	0 .047

### Flushing-related indicators

Significant differences were observed in flushing time (F = 12.34, P < 0.001) and flushing volume (F = 8.76, P = 0.0003). The preheating group demonstrated the shortest flushing time (39.10 ± 3.46 h) and the lowest flushing volume (40.99 ± 3.49 L), both significantly lower than those of the hypothermia group (55.85 ± 7.10 h; 52.80 ± 6.99 L) and mild hypothermia group (47.62 ± 0.46 h; 46.79 ± 1.12 L). Bladder spasm duration also differed significantly among groups (F = 4.56, P = 0.012), with the preheating group showing the shortest duration. The time required for irrigation fluid to clear was slightly shorter in the preheating group, but the difference was not statistically significant (F = 2.13, P = 0.120) (Table 1).

### Laboratory and urine-related indicators

Laboratory indices revealed clear differences among groups, underscoring the impact of irrigation fluid temperature on coagulation and endothelial function (Table 2).

**Table 2:** Comparison of urine-related indicators among the three groups of patients. Note: * It was statistically significant compared with before intervention (*P < 0.05); It was statistically significant compared with the Preheating group (#P < 0.05).

	Hypothermia group (30)		Mild hypothermia group (30)		Preheating group (30)	
	Before<br>intervention	After <br>intervention	Before<br>intervention	After <br>intervention	Before<br>intervention	After <br>intervention
Red blood cell<br>count in urine (/μL)	188 .85 ± 37 .10	29 .62 ± 6.46 *#	183 .10 ± 13 .46	23 .38 ± 7 .34 *#	177 .62 ± 19 .46	6 .10 ± 1 .46 *
APTT (s)	24 .80 ± 6 .99	28 .79 ± 1.12 *#	25 .99 ± 3 .49	26 .12 ± 2 .56	25 .79 ± 1 .12	24 .99 ± 3 .49
PT (s)	8.40± 7.11	9 .21 ± 1 .82	8 .46 ± 5 .38	8 .24 ± 2 .12	9 .21 ± 0 .82	9 .46 ± 0 .38
PLT (10^9^/L)	210 .91 ± 27 .26	171.19± 21.31*#	209 .10 ± 24 .42	191.03± 1 .23*#	208 .42 ± 32 .56	204 .08 ± 30 .34
Pagt(%)	59 .25 ± 7 .45	39 .31 ± 1.13 *#	59 .96 ± 15 .45	41 .16 ± 11 .13*#	61 .49 ± 16 .28	60 .37 ± 14 .54
ET-1	242.98± 7 .51	219 .93 ± 6 .36 *	215 .53 ± 6 .56	217 .97 ± 0 .42 #	214.98± 17.51	187 .93 ± 26 .36 *

#### Urinary red blood cells (RBCs):

Postoperative urinary RBC counts decreased significantly in all groups (P < 0.05). The greatest reduction was observed in the hypothermia group (29.62 ± 6.46 cells/μL) and mild hypothermia group (23.38 ± 7.34 cells/μL), both significantly greater than that in the preheating group (6.10 ± 1.46 cells/μL; P < 0.05). These findings indicate more pronounced hematuria following hypothermic irrigation.

#### Activated partial thromboplastin time (APTT):

Postoperative APTT was significantly prolonged in the hypothermia group (28.79 ± 1.12 s) compared with baseline and with the other two groups (P < 0.05). The preheating group maintained stable APTT values (24.99 ± 3.49 s), suggesting preserved intrinsic coagulation pathway function.

#### Prothrombin time (PT):

No significant postoperative changes in PT were observed across groups (P > 0.05), indicating that irrigation temperature primarily affected intrinsic rather than extrinsic coagulation pathways.

#### Platelet count (PLT):

Platelet counts decreased significantly in the hypothermia (171.19 ± 21.31 X 10^9^/L) and mild hypothermia groups (191.03 ± 1.23 X 10^9^/L) compared with the preheating group (204.08 ± 30.34 X 10^9^/L; P < 0.05). The magnitude of platelet decline was most marked in the hypothermia group, highlighting hypothermia-associated platelet consumption or dysfunction.

#### Platelet aggregation rate (Pagt):

Platelet aggregation capacity was markedly impaired in the hypothermia (39.31 ± 1.13%) and mild hypothermia groups (41.16 ± 11.13%), in contrast to the preheating group which maintained near-normal aggregation (60.37 ± 14.54%; P < 0.05). These results suggest that hypothermia compromises platelet functionality in addition to reducing platelet count.

#### Endothelin-1 (ET-1):

Plasma ET-1 levels decreased significantly in the hypothermia (219.93 ± 6.36 pg/mL) and preheating groups (187.93 ± 26.36 pg/mL) (both P < 0.05 vs. baseline), with the largest reduction observed in the preheating group. The mild hypothermia group showed no significant ET-1 change (217.97 ± 0.42 pg/mL). These findings indicate that hypothermia may blunt endothelial response, whereas preheating enhances endothelial protection and reduces vasoconstrictor burden.

Overall, these laboratory data demonstrate that hypothermia during TURP is associated with impaired coagulation enzyme activity, reduced platelet count and function, and altered endothelial homeostasis, while preheating effectively preserves hemostatic balance.

### Safety evaluation

The incidence of adverse reactions, including chills, shivering, restlessness, bladder spasm, and coagulation dysfunction, was highest in the hypothermia group (33.33%), followed by the mild hypothermia group (23.33%), and lowest in the preheating group (6.66%). The difference among groups was statistically significant (χ^2^ = 6.538, P = 0.038) (Table 3).

**Table 3:** Analysis of Adverse Reactions in the three groups of patients.

	Fear of cold	Shiver	restlessness	Bladder spasm	Coagulation<br>disorders	Adverse reactions
Hypothermia group<br>(30)	3 (10)	2 (6 .66 )	1 (3 .33 )	2 (6 .66 )	2 (6 .66 )	10 (33 .33 )
Mild hypothermia <br>group (30)	2 (6 .66 )	1 (3 .33 )	2 (6 .66 )	1 (3 .33 )	1 (3 .33 )	7 (23 .33 )
Preheating group<br>(30)	1 (3 .33 )	0	1 (3 .33 )	0	0	2 (6 .66 )
* χ^2^*						6 .538
* P *						0 .038

## Discussion

This study demonstrated that the temperature of irrigation fluid significantly affects coagulation function and endothelial homeostasis in patients undergoing transurethral resection of the prostate (TURP). Specifically, hypothermic irrigation (24-26°C) was associated with a notable prolongation of activated partial thromboplastin time (APTT), substantial reductions in platelet count (PLT) and platelet aggregation rate (Pagt), as well as alterations in plasma endothelin-1 (ET-1) levels. Conversely, patients who received preheated irrigation (36-37°C) exhibited more stable laboratory profiles. These findings suggest that the thermal conditions of irrigation fluids exert direct effects on hemostasis and endothelial regulation, independent of their influence on perioperative clinical variables.

The observed extension of activated partial thromboplastin time (APTT) in the hypothermia group underscores the vulnerability of the intrinsic coagulation pathway to temperature variations. Reduced temperatures decrease the enzymatic activity of factors VIII, IX, XI, and XII, thereby impairing thrombin generation and resulting in delayed clot formation. Conversely, prothrombin time (PT), indicative of the extrinsic pathway, remained largely unaffected, indicating that hypothermia induced by irrigation selectively impairs intrinsic rather than extrinsic coagulation mechanisms. This distinction is critical, as dysfunction of the intrinsic pathway is closely linked to perioperative bleeding tendencies [5] [6] [7].

The biology of platelets is markedly affected by the temperature of irrigation. Under hypothermic conditions, both platelet counts and aggregation rates experience a significant decline, indicative of not only increased consumption but also functional inhibition. Hypothermia has been demonstrated to impair the activation of glycoprotein IIb/IIIa receptors, decrease thromboxane A_2_ synthesis, and disrupt actin polymerization, all of which contribute to impaired platelet aggregation. The observed reduction in aggregation capacity in this study aligns with previous in vitro findings that hypothermia modifies platelet membrane fluidity and signaling pathways [8]. The combined decline in PLT and Pagt underscores the dual quantitative and qualitative platelet dysfunction associated with cold irrigation.

In addition, ET-1 dynamics provided insight into the endothelial response to temperature modulation. ET-1, a potent vasoconstrictor and biomarker of endothelial activation, decreased significantly in both hypothermia and preheating groups but with divergent implications. In the preheating group, the reduction may reflect protective downregulation of vasoconstrictor tone and alleviation of endothelial stress. Conversely, the modest decline in the hypothermia group may indicate impaired endothelial secretory function, consistent with prior evidence that cold exposure disrupts calcium signaling and endothelial barrier integrity [9] [10]. Thus, ET-1 serves as an important biochemical correlate of vascular dysfunction under thermal stress.

From the perspective of laboratory medicine, these findings hold significant clinical relevance as they identify potential biomarkers for predicting bleeding complications. Specifically, prolonged activated partial thromboplastin time (APTT), reduced platelet counts, impaired platelet aggregation rates, and altered endothelin-1 (ET-1) profiles may serve as early indicators of hypothermia-induced coagulopathy. Integrating these parameters into perioperative monitoring protocols could enable timely interventions, such as active warming strategies, platelet transfusion, or targeted supplementation of coagulation factors.

Although bladder spasm and flushing outcomes were also affected by irrigation temperature, their interpretation primarily reflects clinical phenomena. The strength of this study lies in the detailed evaluation of laboratory indices that reveal the biochemical mechanisms underlying hypothermia-induced bleeding. These results underscore the importance of integrating coagulation and endothelial biomarkers into perioperative research, particularly for elderly patients and those with cardiovascular or hematologic vulnerabilities [11].

Despite the robust findings, this study has several limitations. First, the relatively small sample size (n=30/group) may limit the generalizability of our conclusions, particularly for subgroup analyses. Second, the single-center design introduces potential selection bias and restricts the diversity of patient demographics. Third, the absence of long-term follow-up prevents assessment of delayed bleeding events or endothelial function recovery. Fourth, the use of 5% glucose as irrigation fluid might interact with temperature effects, which warrants validation in balanced salt solutions. Finally, while we controlled core temperature, unmeasured confounders could influence coagulation dynamics.

## Conclusions

This study shows that the temperature of irrigation fluid significantly affects coagulation and endothelial biomarkers in TURP patients. Cold irrigation fluid (24-26°C) led to prolonged APTT, reduced platelet count and function, and altered ET-1 levels, indicating impaired hemostasis and endothelial dysfunction. Conversely, preheated irrigation maintained coagulation stability and endothelial protection. These results suggest that routine lab parameters like APTT, PLT, Pagt, and endothelial markers such as ET-1 could be useful for detecting hypothermia-induced coagulopathy and predicting bleeding risk during surgery. The study emphasizes the need for integrating lab diagnostics into perioperative monitoring and thermal management. Future research should confirm the predictive value of these biomarkers and explore their role in improving patient outcomes through standardized protocols.

## Dodatak

### Conflict of interest statement

All the authors declare that they have no conflict of interest in this work.
